# An engineered glove for investigating the neural correlates of finger movements using functional magnetic resonance imaging

**DOI:** 10.3389/fnhum.2015.00503

**Published:** 2015-09-14

**Authors:** Laura Bonzano, Andrea Tacchino, Luca Roccatagliata, Matilde Inglese, Giovanni Luigi Mancardi, Antonio Novellino, Marco Bove

**Affiliations:** ^1^Department of Neuroscience, Rehabilitation, Ophthalmology, Genetics, Maternal and Child Health, University of GenoaGenoa, Italy; ^2^Magnetic Resonance Research Centre on Nervous System Diseases, University of GenoaGenoa, Italy; ^3^Department of Experimental Medicine, Section of Human Physiology, University of GenoaGenoa, Italy; ^4^Department of Health Sciences, University of GenoaGenoa, Italy; ^5^Department of Neurology, Radiology, Neuroscience, Icahn School of Medicine at Mount SinaiNew York, NY, USA; ^6^Research and Development Unit, ETT S.p.A.Genoa, Italy

**Keywords:** engineered glove, finger movements, fMRI, motor performance, MR compatibility

## Abstract

Objective measurement of concomitant finger motor performance is recommended for functional magnetic resonance imaging (fMRI) studies investigating brain activity during finger tapping tasks, because performance modality and ability can influence the selection of different neural networks. In this study, we present a novel glove system for quantitative evaluation of finger opposition movements during fMRI (called Glove Analyzer for fMRI, GAF). Several tests for magnetic resonance (MR) compatibility were performed concerning magnet forces, image artifacts and right functioning of the system. Then, pilot fMRI of finger opposition tasks were conducted at 1.5T and 3T to investigate the neural correlates of sequences of finger opposition movements with the right hand, with simultaneous behavioral recording by means of GAF. All the MR compatibility tests succeeded, and the fMRI analysis revealed mainly the activation of the left sensorimotor areas and right cerebellum, regions that are known to be involved in finger movements. No artifactual clusters were detected in the activation maps. At the same time, through the parameters calculated by GAF it was possible to describe the sensorimotor strategy adopted by the subjects during the required task. Thus, the proposed device resulted to be MR compatible and can be useful for future fMRI studies investigating the neural correlates of finger opposition movements, allowing follow-up studies and comparisons among different groups of patients.

## Introduction

The ability to perform finger opposition movements, rapidly and independently, is crucial in daily-life activities and has been the topic of a large number of investigations, ranging from speed and accuracy of simple motor tasks (e.g., single finger tapping) to complex motor exercises (e.g., bimanual finger opposition movement sequences in synchrony with external cues). Finger movement sequences are commonly used in motor learning tasks (Karni et al., 1995); finger motor tasks can be based on single finger tapping (Grafton et al., 1998) or sequences of finger presses (Walker et al., 2003; Plewnia et al., 2006; Balas et al., 2007; Sheth et al., 2008; Wilhelm et al., 2008).

Brain activity during hand movements can be investigated by functional magnetic resonance imaging (fMRI); finger tapping tasks can be easily performed inside an magnetic resonance (MR) scanner, however many studies have been conducted without behavioral measurements during the fMRI sessions. Indeed, quantitatively measuring movement performance is crucial in fMRI experimental designs involving motor tasks, since brain activity has been shown to correlate with movement-dependent changes (Ashe and Ugurbil, 1994; Rao et al., 1996; Martinez et al., 2014). Healthy subjects are able to generate and maintain self-paced rhythmic movements and to synchronize them with external signals, and the rate of the external cue determines the selection of different neural mechanisms. Also, correlating motor performance with changes in cortical organization during learning of finger movement sequences can become a useful tool to investigate sensory, motor and cognitive systems in healthy subjects and subjects suffering from neurological diseases. Recently, we demonstrated that the subjects achieving greater movement rate increase in a finger motor learning protocol were those showing stronger resting state functional connectivity of the left primary motor cortex and supplementary motor area with the right lobule VIII of the cerebellum (Bonzano et al., 2015).

In this context, there is the need of MR-compatible devices, which can be used during fMRI not introducing risks for patient safety and image quality. Research labs are very active in designing prototypes of innovative MR-compatible movement measurement systems based on accelerometers and gyroscopes (Schaechter et al., 2006; Kim et al., 2013), or video tracking (Hauptmann et al., 2009), or video and force sensors (Hou et al., 2005; Rogers et al., 2010). However, relatively few fMRI motor studies have recorded performance on-line because the signal from a motion sensor can be grossly contaminated by noise induced from the scanning environment, and because presence of a sensor during fMRI can result in image artifacts. Some examples designed to overcome this problem have been the use of a non-ferromagnetic (Ehrsson et al., 2001; Cramer et al., 2002) or hydraulic (Liu et al., 2000) force transducer to measure handgrip force, and an accelerometer (Morgen et al., 2004) and electrogoniometer (Carey et al., 2002) to monitor movement of a single finger during fMRI. Further, the angular components of finger movements were evaluated during fMRI using MicroElectro-Mechanical System (MEMS) gyroscopes (Schaechter et al., 2006). Recently, the possibility to use an optoelectronic motion capture system during an fMRI study was explored (Casellato et al., 2010). Nowadays, the commercially available MR-compatible tools are mainly based on response boxes and, usually, they are not able to measure the kinematic parameters to describe finger motor performance strategy: for instance they can be used to record response time to a given stimulus or to assess the ability of a subject to reproduce a finger motor sequence by presses, usually recording the button pressed without any information regarding the time of contact. To our knowledge the only example of assessment of finger movement kinematics by using an MR-compatible keypad was performed by Orban et al. (2011).

Recently, we designed a system based on a simple and comfortable wearable glove to record the kinematics of finger opposition movement sequences in unimanual or bimanual motor tasks. A software package records the finger touches with the thumb and provides semi-automatic analysis tools for calculating both spatial and temporal parameters of motor sequences (Bove et al., 2007). The first version of this device was called Glove Analyzer System (GAS; ETT S.p.A., Italy); it has been used for several research studies conducted on healthy subjects and patients affected by neurological diseases, such as multiple sclerosis (MS; Bove et al., 2007, 2009; Bonzano et al., 2008, 2011a,b, 2013a, 2014). In particular, the first study (Bove et al., 2007) was conducted on healthy subjects and allowed us the investigation of the changes in different finger motor parameters with increased task complexity and speed. This constituted an important reference for the choice of the experimental protocol to adopt in the following studies. Then, we conducted some studies on MS and correlated specific impairment to localized brain damage to investigate the role of different neural structures (e.g., the role of the corpus callosum in bimanual coordination and in the intermanual transfer of learning). Importantly, we conducted a study on a large cohort of subjects with MS in comparison to a group of healthy subjects, in order to assess the reproducibility of the finger motor parameters and to find out the parameters which contributed independently to differentiate the two groups (Bonzano et al., 2013a). This study demonstrated that this simple, quantitative, objective method measuring finger motor performance could be used to define a score discriminating healthy controls and patients with MS, even with very low disability, and it is crucial for monitoring the disease course and the treatment effects starting from the early phase of the disease.

Here, we present a new system called Glove Analyzer for fMRI (GAF) to analyze finger opposition sequences of different complexities in fMRI environment, thus allowing the correlation between brain activity and the kinematics parameters assessing the accuracy of finger opposition movements acquired simultaneously.

This new prototype of the glove system and the compatibility testing procedures performed are presented together with the results from the first fMRI experiments carried out using both a 1.5T and a 3T scanner.

## Methods

### Instrumentation

**Figure 1:**
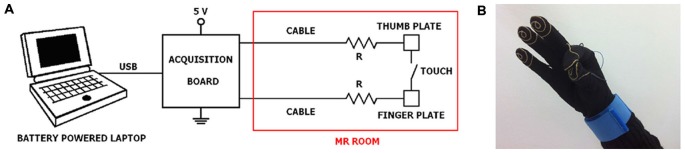
**Experimental set-up. (A)** General connection scheme of laptop, acquisition board and engineered glove system; **(B)** a glove prototype with an example of thumb-to-index finger contact. It should be noted that a subdivision of the equipment in the portions that can be positioned outside and inside the MR room is required.

The system consists of three distinct parts: a wearable engineered glove, an acquisition board, and a laptop with the necessary software installed (Figure 1).

#### Glove Prototype

The engineered glove is built on a Lycra glove, easy to wear and not exerting constraints during finger movements. Five thin wires of gold are sewn as a spiral on the palmar surface of the distal phalanxes of the glove, like fingerprints, in order to record the contact during opposition movements between the thumb and another finger. Each spiral is connected through its own wire and a specific connector in brass gilt to a bracelet in tissue with a Velcro closure. Tinsel interlaced with strands in Ag/Cu was used as wire. The total distance from the spiral to the bracelet is 30 cm. Further, a security MR-compatible resistor (12 kΩ) is placed at 1 cm far away the spiral. From the bracelet, the five signals corresponding to the five fingers reach the acquisition board (USB-1208FS, Measurement Computing, USA), positioned outside the MR room, through a multipolar cable ending with a 9-pin RS-232 connector.

#### Software

The program, i.e., “Glove”, used for recording and analysis of the data was developed with the Microsoft Visual Studio 2013, .NET Framework 4.5; the program was written in C# exploiting the Windows Presentation Foundation WPF for desktop application development platform. The software package is provided with an embedded “Volante” database,^1^ which hosts personal data, tasks and protocols. It runs under Microsoft Windows 7 (or more) Operating System. Glove also integrates the acquisition board drivers and configuration features.

Several protocols with different features can be implemented. First, it is possible to leave the movement free, without time constraints, or to deliver a cue to guide the movement. Thus, when no external events pace movements subjects can be instructed to perform a sequence of finger opposition movements at their comfortable velocity (spontaneous velocity (SV) condition) or maximal velocity (MV condition). On the other hand, subjects can be asked to execute the finger opposition movements by tapping in synchrony with an external pace (Metronome condition): acoustic or visual cues can be set at a chosen rate. A specific sequence of finger opposition movements (e.g., opposition of the thumb to the index finger only, or to the index, medium, ring and little fingers in a predetermined order) can be chosen for an experimental session; the sequence needs to be set before the session to allow the software to automatically execute the match with the streaming of touches of the subject (Bove et al., 2007; Bonzano et al., 2013a). The task can be performed continually for a time interval set by the experimenter, with the dominant or non-dominant hand (unimanual condition), or with the two hands simultaneously (bimanual condition). Thus, experimental protocols of different levels of complexity can be adopted according to the study purposes.

At the end of the recording session, the system is able to perform the analysis of several parameters describing finger opposition movements. The analysis is performed on the sequences correctly executed within a task; mean value, standard deviation, standard error, and coefficient of variation are automatically calculated for each parameter described in the following.

Spatial accuracy can be objectively described by means of Error Number (EN), i.e., the absolute number of errors performed, percentage of correct sequences (%CORR_SEQ), i.e., the percentage of sequences performed correctly over the total number of sequences in the recording session. Sensorimotor strategy in its timing aspects can be evaluated through: Touch Duration (TD), i.e., the contact time between the thumb and another finger (measured in ms), Inter Tapping Interval (ITI), i.e., the time interval between the end of a thumb-to-finger contact and the beginning of the subsequent contact in the finger motor sequence (measured in ms), movement rate (RATE), computed as (1/(TD + ITI)) × 1000 (measured in Hz; it represents the number of touches per second), ratio TD/ITI, i.e., adimensional parameter indicating the relative movement time spent in finger discrimination. Also, to describe temporal accuracy for the protocols with an external pace (Metronome condition) the following parameters were defined: Timing Error (TE): i.e., the time interval between the touch onset and the corresponding acoustic or visual cue (TE is measured in ms; when the touch precedes the cue TE is negative, when the touch follows the cue TE is positive), percentage of advance movements (%ADV_MOV), i.e., the percentage of touches preceding the cue over the total number of correct touches. For bimanual trials, bimanual coordination can be assessed by means of the Inter Hand Interval (IHI), calculated as the absolute time difference between the touch onset occurring in the left hand and the corresponding touch in the right hand (measured in ms): the larger the IHI value, the more severe the impairment in bimanual coordination (Bonzano et al., 2008).

### Compatibility Study

A device can be considered MR compatible if and only if: (i) its presence in the MR magnet room does not pose an increased safety risk to the patient or other personnel; (ii) it performs its intended function when used in conjunction with the MR system in a safe and effective manner; (iii) its use in conjunction with the MR scanner does not adversely impact the function of the scanner.

**Figure 2:**
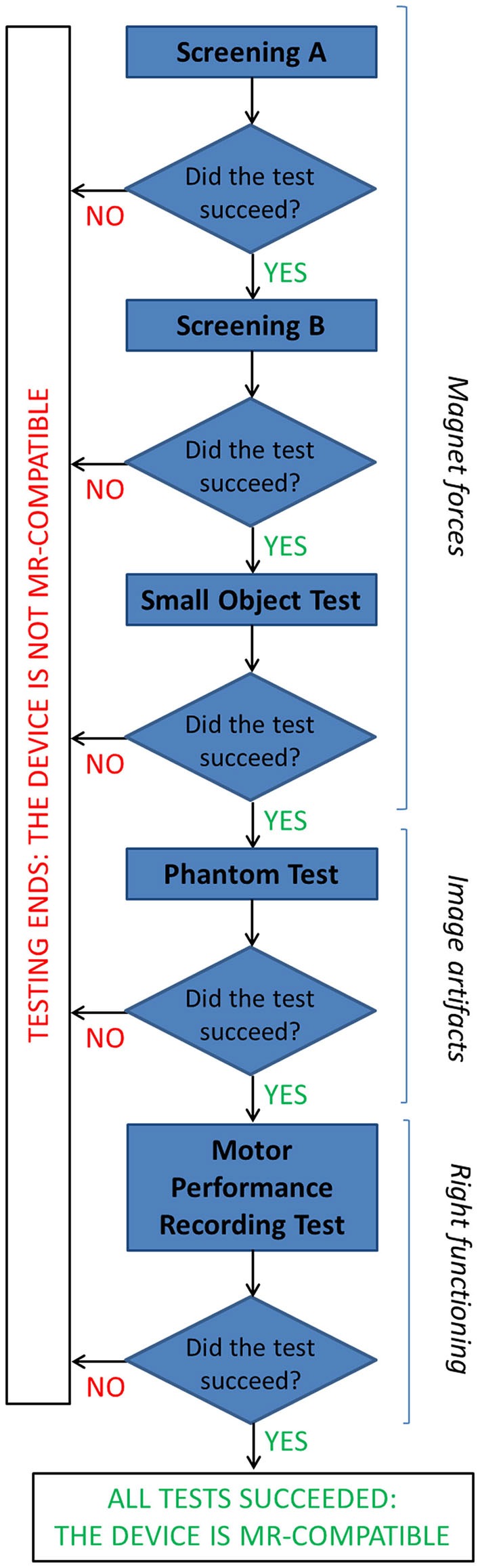
**The flow chart describes the tests performed to assess MR compatibility of the GAF system (details are reported in the text)**.

Different compatibility tests were performed at 1.5T to ensure no influence of the device on patient safety and image artifacts, and right functioning of the device (that could be altered by the large static magnetic field, rapidly changing magnetic field and radio frequency energy). The adopted flow chart is represented in Figure 2.

#### Magnet Forces

The first concern relates to the magnet forces: the device must not be attracted to the magnet with sufficient force as to be a projectile and it must not experience sufficient torque as to be a possible source of injury.

Prior to entering the MR procedure room, a screening test on GAF was conducted outside. To test for magnetic attraction, a pocket-size hand magnet with plastic handle (Edmund Scientific 25lb. Pull SN #40847) was suspended on a string, and a sheet of paper was put on the wall in order to assess its perpendicularity to the floor. Then the device was slowly brought into contact with the magnet. This test is passed if no magnetic attraction between the two objects is observed (“Screening A”). To detect any magnetic force or torque, outside the MR room the device was placed in a Plexiglas box on a sheet of paper on which an outline of the device was drawn to indicate its initial position. Then the box was closed and the lid secured; the box was placed on the patient table, which was moved to take the box with the device to the magnet isocenter, then it was returned to the starting position. The test is passed if the position of the device relative to its outline has not changed, including rotation (“Screening B”). A second test to determine the magnetic force and torque required a deflection meter: while outside the procedure room GAF was placed into a mesh bag, hanged from the center of a semi-circle goniometer with a string, and slowly carried into the procedure room toward the magnet. The angle of deflection of the string is monitored to assess absence of movement, otherwise the test must be stopped and the device removed from the procedure room. Then, this set-up was placed at the top of the magnet opening, always monitoring the deflection angle (“Small Object Test”).

#### Phantom Test

To evaluate the presence of noise and image artifacts related to the presence of the device, different imaging tests were performed by using an MRI Phantom. As a general rule, in these tests the best obtainable result is to get the same level of performance both with and without the device.

**Figure 3:**
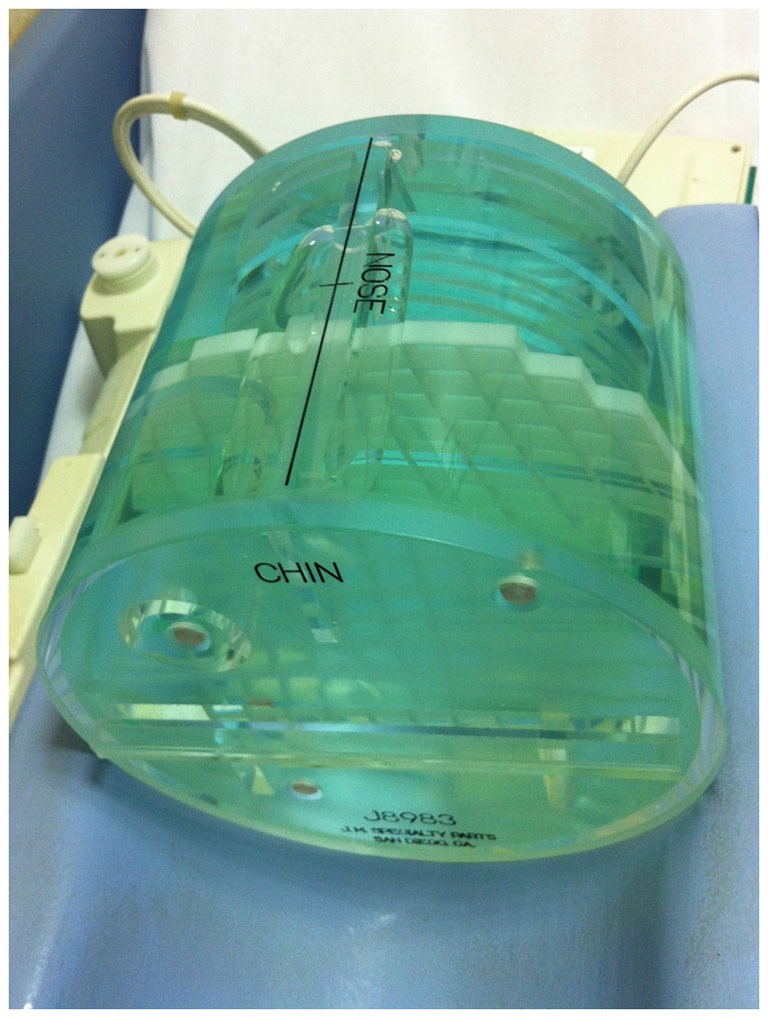
**A photograph of the ACR MRI Phantom in the head coil used to evaluate possible image artifacts due to the presence of the device**.

In details, the American College of Radiology (ACR) MRI Phantom was adopted (Figure 3). This consists of a hollow cylinder of acrylic plastic closed at both ends, ensuring the absence of ferromagnetic materials, filled with a solution of nickel chloride (10 mM) and sodium chloride (75 mM). The outside of the Phantom has the words “NOSE” and “CHIN” etched into it as an aid to orienting the Phantom for scanning, as if it were a head. Inside the Phantom are several structures designed to facilitate a variety of tests of scanner performance (Phantom Test Guidance 2005 for the ACR MRI Accreditation Program).^2^

The ACR MRI Phantom was scanned in the head coil with the cylindrical Phantom aligned as a head. The center of the phantom was placed in the center of the head coil and aligned with the positioning indicator light so that it resulted to be in the isocenter of the scanner. The imaging protocol included a T2-weighted sequence (23 slices; slice thickness = 5 mm; gap between slices = 1 mm; TR = 6300 ms; TE = 123.9 ms; FOV = 260 × 260 mm; matrix = 256 × 256) and an echo-planar (EPI) sequence (23 slices; slice thickness = 5 mm; gap between slices = 1 mm; TR = 3000 ms; TE = 40 ms; FOV = 260 × 260 mm; matrix = 64 × 64; 43 volumes) in a 1.5T MR system (Signa Excite, General Electric Healthcare, WI, USA). The first 3 volumes of EPI sequences were discarded to allow for steady-state magnetization.

We performed different tests for image quality in different experimental conditions to assess the effects of the presence of the device under consideration. In fact, for each sequence (EPI and T2), three different acquisitions were performed at different conditions: (a) “No GAF” (baseline condition), i.e., imaging tests were performed on the phantom without the presence of any other devices; (b) “GAF in FOV”, i.e., tests were carried on the phantom with the GAF system placed inside the field of view (on the top of the phantom); (c) “GAF out of FOV”, i.e., tests were performed on the phantom with the GAF system placed outside the FOV (at a distance of about 70 cm as the averaged distance between the subject’s head and hand).

The acquired images were analyzed with ImageJ software through *ad hoc* developed macros according to the specific tests.

The estimation of the signal-to-noise ratio (SNR) was determined on both T2 and EPI images, whilst the following quantitative tests were conducted only on the T2-weighted scans (these tests were meaningful only for structural sequences): “Geometric accuracy”, “Slice thickness accuracy”, “Low-contrast object detectability”.

SNR was assessed with the “Five ROIs analysis test”: five rectangular ROIs were delineated on a homogeneous slice of the Phantom, one on the top of the image, three in the center and one at the bottom. The mean signal intensity value S and the standard deviation value SD were calculated in each ROI and the SNR was derived as in Eq. (1).

(1) $$\text{SNR =}\sum {\text{SNR}}_{\text{i}}\text{/5}$$

where SNR_i_ is the SNR of each ROI given by Eq. (2).

(2) $${\text{SNR}}_{\text{i}}{\text{ = S}}_{\text{i}}{\text{/SD}}_{\text{i}}$$

The same ROIs were chosen for the three experimental conditions and the SNR values were compared among them. Repeated measures-ANOVA (RM-ANOVA) was performed to investigate possible differences in SNR among the three conditions: No GAF, GAF in FOV, GAF out of FOV. RM-ANOVAs were corrected for potential violations of sphericity, adjusting their degrees of freedom using the Greenhouse–Geisser correction.

**Figure 4:**
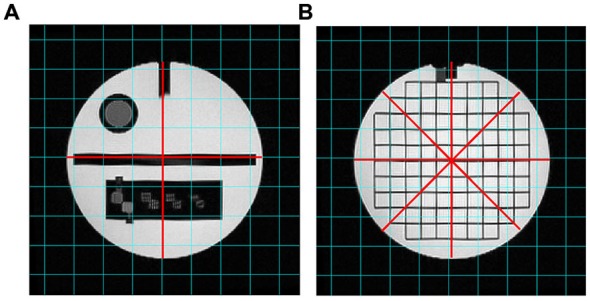
**T2-weighted images analyzed for the “Geometric accuracy” test**. The red lines indicate the diameters to be measured on slice 5 **(A)** and slice 12 **(B)**.

The “Geometric accuracy test” assessed the accuracy with which lengths were represented in the image. It consists in making length measurements between readily identified locations in the Phantom and comparing the results with the known values for those lengths. A failure means that dimensions in the image differ from the true dimensions substantially more than is usual for a properly functioning scanner. Measurements for this test were performed on two different slices of the T2-weighted scans (i.e., slice 5 and slice 12), as represented in Figure 4.

The diameter of the Phantom on slice 5 was measured in two directions: top-to-bottom and left-to-right, whilst on slice 12 in four directions: top-to-bottom, left-to-right and both diagonals. The length measurements were then compared with the known values of the distances in the Phantom (inside end-to-end length = 148 mm; inside diameter = 190 mm). The test succeeds if all measured lengths are within ± 2 mm of their true values.

**Figure 5:**
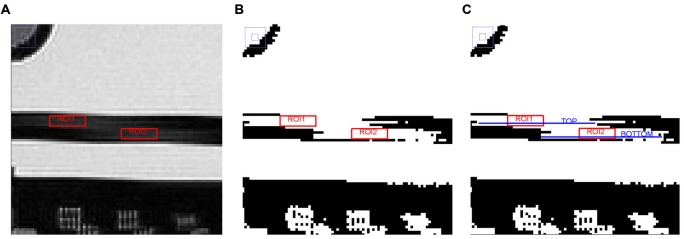
**Different steps adopted in the image analysis on slice 5 for the “Slice thickness accuracy test” (see the text for further details). (A)** Magnified region of slice 5 showing slice thickness signal ramps with ROIs placed for measuring average signal in the ramps, **(B)** Slice 5 after setting the new display window level value, and **(C)** Slice 5 with TOP and BOTTOM elements represented by linear ROIs.

The “Slice thickness accuracy test” assessed the accuracy with which a slice of specified thickness is achieved. The prescribed slice thickness is compared with the measured slice thickness (the implications of a failure can lead to incorrect image contrast and low SNR). Figure 5 shows the different steps adopted in the procedure.

The lengths of two signal ramps in slice 5 were measured. Slice thickness was represented by the dark rectangle in the slice center, the two ramps were the lighter streaks inside of it. The length of the signal ramps were measured by displaying slice 5 magnified by a factor of 4, keeping the slice thickness fully visible on the screen. Then, a rectangular ROI was placed in the middle of each signal ramp; the mean value of each ROI was calculated and the two obtained values were averaged together, achieving a number approximating the mean signal in the middle of the ramps. The average ramp signal was halved and the result used as the display window level value, whereas the window width value was set to zero. The length of the top and bottom ramps were determined by measuring the perimeter of two linear ROIs placed in the ramps (defined as “TOP” and “BOTTOM”, respectively). Slice thickness was calculated by the following Eq. (3):

(3) SLICE THICKNESS = 0.2 × (TOP × BOTTOM)/(TOP + BOTTOM)

The measured slice thickness should be 5.0 ± 0.7 mm.

**Figure 6:**
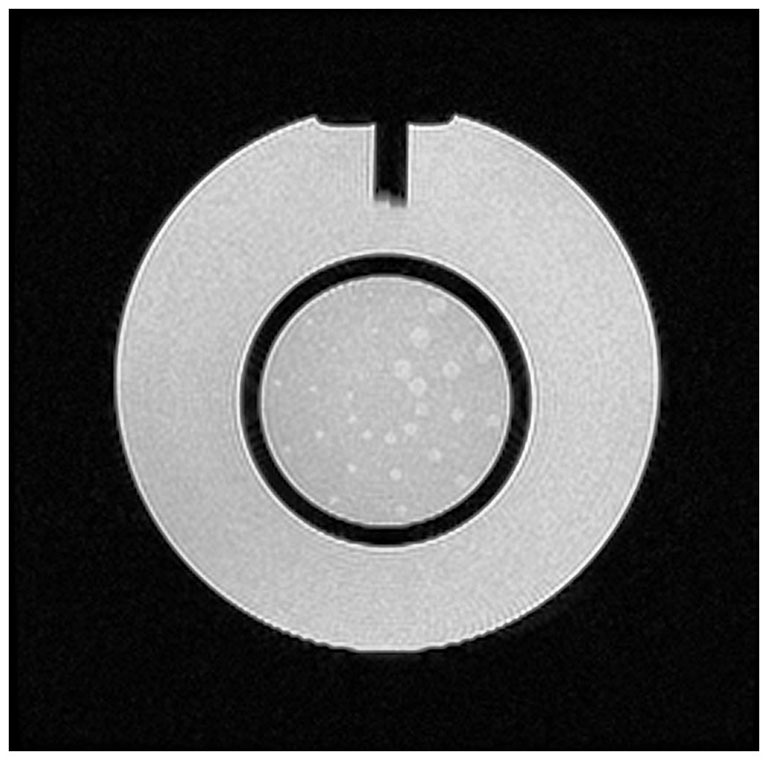
**Slice 22, with low-contrast objects of varying size and contrast for the “Low-contrast object detectability” test**.

The “Low-contrast object detectability test” assessed the extent to which objects of low contrast were discernible in the images. For this purpose the Phantom has a set of low-contrast objects of varying size and contrast (visible on slice 22, displayed in Figure 6): these are rows of small disks, radiating from the center of a circle like spokes in a wheel, with 10 spokes per circle and 3 disks per spoke. All the disks in a given spoke have the same diameter. Starting at the 12 o’clock position and moving clockwise, the disk diameter decreases progressively from 7.0 mm at the first spoke to 1.5 mm at the tenth spoke.

The measurements for this test consist of counting the number of complete spokes seen. The display window width and level settings were adjusted for the best visibility of the low-contrast objects. A spoke is considered complete only if all three of its disks are discernible. The test is passed if a total score of at least 9 spokes is achieved. If the images are free of ghosts, and the slices are positioned accurately, a failure of this test is most likely due to inadequate SNR in the image. Also, the number of disks was evaluated both by using ImageJ applications and by naked eye. On ImageJ objects were isolated through the application of a threshold and the number of disks was counted by choosing a range area value. The right number of disks to be found is 30.

#### Right Functioning

Before moving to the first fMRI experiments, the device was tested inside the MR room with a researcher close to the scanner performing simple sequences of finger opposition movements, to assess the ability of the system to correctly record the touches and calculate the finger motor performance parameters without any influence of the MR environment (“Motor performance recording test”).

Following the tests at 1.5T, we moved to the 3T environment.

The magnetic attraction tests were performed before introducing the device in the MR room and bringing it close to the scanner. Then, SNR was assessed on EPI images before implementing the fMRI protocol: axial slices of a cylindrical homogeneous MRI phantom were acquired (TIM TRIO, Siemens Medical Solutions, Germany; 36 slices; slice thickness = 3 mm; gap between slices = 0.75 mm; TR = 3000 ms; TE = 30 ms; FOV = 192 × 192 mm; matrix = 64 × 64; 20 volumes plus three dummy volumes) in the No GAF condition and in the GAF out of FOV condition, and SNR was calculated with the “Five ROIs analysis test”. Last, the proper functioning of the device was assessed.

### fMRI Study

After assessing safety and compatibility, preliminary fMRI experiments were performed on 10 healthy subjects with a 1.5T and then on one healthy subject on a 3T MR scanner with the use of the GAF system to measure motor performance, in comparison with an fMRI acquisition without GAF. Informed consent was obtained according to a procedure approved by the local ethics committee (Comitato Etico Regione Liguria, IRCCS Azienda Ospedaliera Universitaria San Martino—IST, Genoa, Italy).

The motor task consisted in the repetition of a finger motor sequence with the right (dominant) hand, i.e., opposition of the thumb to the index, medium, ring and little fingers, self-paced at SV. Subjects kept their eyes closed during the whole experimental session to avoid visual cortical activation.

Within each fMRI run, participants had to perform either the described finger motor task (task) or to stay at rest without making any overt movement (rest), according to a boxcar design with two 30 s task periods alternating with two 30 s rest periods.

The fMRI scanning series consisted in axial T2*-weighted single-shot EPI sequences covering the whole brain and were performed with a T/R head coil on the 1.5T scanner (23 slices; slice thickness = 5 mm; TR = 3000 ms; TE = 40 ms; FOV = 260 × 260 mm; matrix = 64 × 64; 43 volumes of which the first three were discarded to allow steady-state magnetization) and with a 16-channel phased-array head coil on the 3T scanner (36 slices; slice thickness = 3 mm; gap between slices = 0.75 mm; TR = 3000 ms; TE = 30 ms; FOV = 192 × 192 mm; matrix = 64 × 64; 60 volumes plus 3 dummy volumes).

SPM5 software (Wellcome Department of Imaging Neuroscience, London, UK) was used for fMRI processing and statistical analysis (Friston et al., 1995). For each subject, after movement correction and slice timing, the functional images were realigned to the first functional image, normalized to the Montreal Neurological Institute (MNI) template brain image using a 12-parameter affine transformation, re-sampled to 2 × 2 × 2 mm^3^ voxels and smoothed with an 8 mm full-width at half-maximum isotropic Gaussian kernel to increase the SNR. A general linear model was used to identify the voxels with task-related signal changes at individual level. Task-related (right hand > baseline) *t* contrast images were created for each subject in each experimental condition (with GAF and without GAF) separately, and the corresponding activation maps were obtained using a height threshold of *p* < 0.05 FWE corrected and minimum cluster size of 20 voxels. Then, paired *t*-tests were performed between the two conditions (with GAF and without GAF).

## Results

### Compatibility Study

The GAF system passed the tests related to magnetic force or torque: the procedure was followed step-by-step moving to the successive test when a test was passed. In the following, the results concerning image quality are presented for each MR sequence and experimental condition.

**Figure 7:**
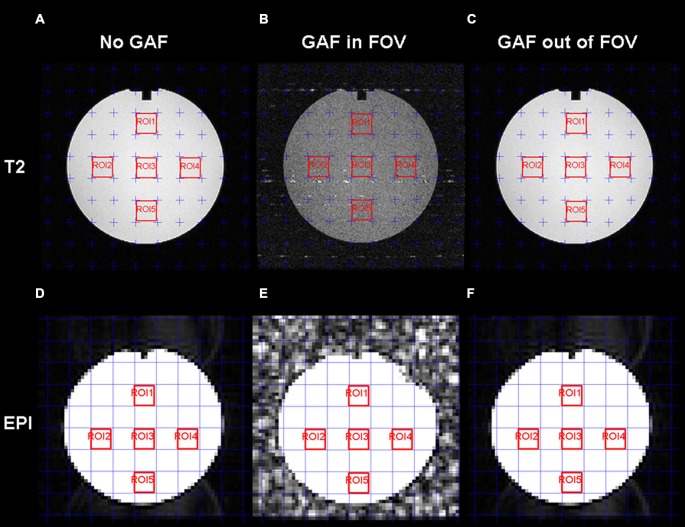
**T2-weighted (A,B,C) and EPI (D,E,F) images with the five ROIs delineated in red for the SNR estimation**. For each series, the three conditions are displayed: No GAF **(A,D)**, GAF in FOV **(B,E)**, and GAF out of FOV **(C,F)**.

Figure 7 shows the images acquired for the estimation of the SNR. From a qualitative point of view, reduced quality is evident on both the T2 and EPI images when the device was placed close to the coil (GAF in FOV condition).

**Figure 8:**
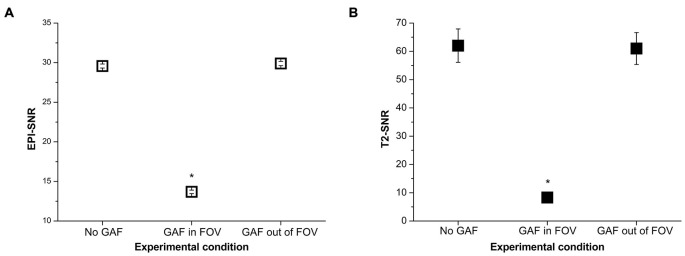
**SNR values (mean ± s.e.) calculated on the EPI (A)** **and T2 (B) images in the different experimental conditions**. *Indicates statistical significance (SNR in the GAF in FOV condition was significantly reduced with respect to the other two conditions).

The mean SNR values calculated in the different conditions are reported in Figure 8. RM-ANOVA on the single ROIs values showed that the SNR measured on EPI images was significantly different among the three different conditions (*F*_(2,390)_ = 5932.30, *p* < 0.0001; *F*_(1.22,237.46)_ = 5932.30, *p* < 0.0001 after Greenhouse-Geisser correction with ε = 0.61). Newman-Keuls *post hoc* test indicated that SNR in the GAF in FOV condition was significantly lower than in the other two conditions (*p* < 0.0001). Conversely, SNR in the GAF out of FOV condition was similar to SNR in the No GAF condition. Comparable results were obtained when calculating the SNR on the T2-weighted images (*F*_(2,8)_ = 83.42, *p* < 0.00001; *F*_(1.15,4.59)_ = 83.42, *p* < 0.001 after Greenhouse-Geisser correction with ε = 0.57), where a significantly reduced SNR was observed in the GAF in FOV condition with respect to the other two conditions (*p* = 0.0002), and no difference was found in the GAF out of FOV condition with respect to the No GAF condition. All these findings indicate that GAF did not compromise image quality in both EPI and T2 sequences when it was positioned at the level of the hand (i.e., about 70 cm far from the head—MRI Phantom), which corresponds to the proper use for which it was developed.

Similar findings were achieved at 3T. Paired *t*-test showed no significant difference in SNR calculated in the middle slice (i.e., 18) between the No GAF and the GAF out of FOV conditions (No GAF: SNR = 49.041 ± 3.349, GAF out of FOV: SNR = 49.037 ± 3.370; *t*_(99)_ = 0.20, *p* = 0.84).

In the Geometric accuracy test, the presence of the glove in the FOV made more difficult to place the linear ROIs; however, even though the image was blurry the diameter results were in the correct range also in this condition (190 ± 2 mm) (Table 1).

**Table 1:** **Results from the Geometric accuracy test (diameter)**.

Condition	Diameter	Measure on slice 5 (mm)	Measure on slice 12 (mm)
**No GAF**	Top-to-bottom	189.88	188.70
	Left-to-right	188.70	189.49
	Diagonal		189.49
	(from left to right)		
	Diagonal		189.44
	(from right to left)		
**GAF in FOV**	Top-to-bottom	189.49	188.30
	Left-to-right	189.09	188.70
	Diagonal		189.47
	(from left to right)		
	Diagonal		188.88
	(from right to left)		
**GAF out of FOV**	Top-to-bottom	190.27	189.88
	Left-to-right	188.70	188.31
	Diagonal		188.64
	(from left to right)		
	Diagonal		189.15
	(from right to left)		

The results obtained for the Slice thickness in the No GAF condition and in the GAF out of FOV condition were correct (4.62 mm and 5.17 mm, respectively), whereas the slice thickness value measured in the GAF in FOV condition was not included in the allowed range (3.82 mm).

The Low-contrast object detectability test revealed that the presence of the glove in the FOV hampered the spokes count, while the number of spokes and disks counted for the other two conditions was correct.

### fMRI Study

**Figure 9:**
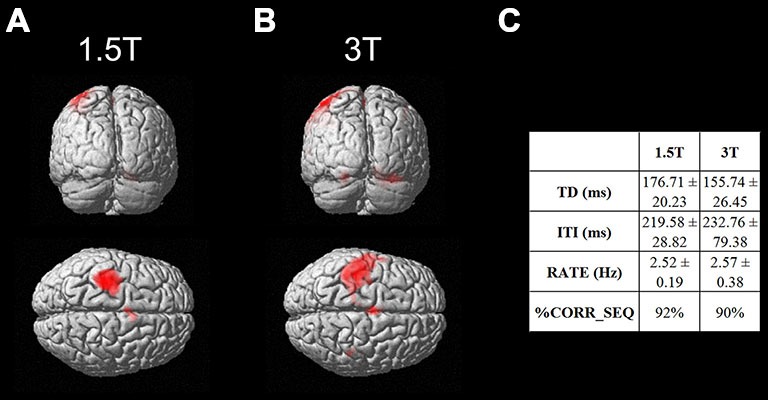
**Brain activation maps related to the finger motor sequence performed with the right hand**. Results are displayed on a rendering surface in the neurological convention (left is left). One representative subject is shown at 1.5T **(A)** and 3T **(B)**, in the “with GAF” condition. In the table **(C)**, the kinematics parameters describing the motor performance are shown (TD: Touch Duration, ITI: Inter Tapping Interval, RATE: mean frequency during the finger motor task, %CORR_SEQ: percentage of correct sequences).

As a first result, the healthy volunteers who underwent the fMRI study showed full feasibility of the procedure and no complaints of heating or any discomfort. Figure 9 shows the imaging results for one representative subject at 1.5T and 3T (a healthy volunteer who underwent the fMRI acquisition with both scanners), in the “with GAF” condition. The corresponding MNI coordinates and statistics are reported in Table 2.

**Table 2:** **Brain areas of activation during the finger motor task with the right hand wearing the glove (see Figure 9) for a representative subject at 1.5 T and 3T**.

Experimental session	Cluster size	Voxel T	Voxel Z	MNI coordinates *x, y, z* (mm)	Laterality	Brain structure
1.5T	1518	9.74	Inf	−34 −22 64	Left	Brodmann area 4
	226	6.34	5.95	24 −52 −22	Right	Cerebellum
		6.18	5.81	14 −56 −14	Right	Cerebellum
	164	5.7	5.4	4 4 68	Right	Brodmann area 6
		5.69	5.4	−2 0 62	Left	Brodmann area 6
3T	2247	15.57	Inf	−42 −22 64	Left	Brodmann area 4
		14.96	Inf	−44 −36 62	Left	Brodmann area 2
		13.89	Inf	−34 −24 70	Left	Brodmann area 6
	857	12.71	Inf	16 −54 −14	Right	Cerebellum
		12.59	Inf	28 −54 −24	Right	Cerebellum
		11.47	Inf	20 −50 −24	Right	Cerebellum
	334	9.21	Inf	−20 −56 −18	Left	Cerebellum
		9.18	Inf	−22 −72 −18	Left	Cerebellum
		7.09	6.42	−22 −48 −28	Left	Cerebellum
	343	8.32	7.31	−2 −6 58	Left	Brodmann area 6
		7.13	6.45	−4 −8 74	Left	Brodmann area 6
	101	7.59	6.79	34 −78 −22	Right	Cerebellum
	94	6.98	6.34	42 −30 42	Right	Brodmann area 2
		6.68	6.1	44 −34 52	Right	Brodmann area 40
		5.5	5.16	46 −22 38	Right	Brodmann area 3
	90	6.62	6.06	−58 −20 16	Left	Brodmann area 40
		6.42	5.9	−60 −16 6	Left	Brodmann area 22

*Coordinates are reported according to the Montreal Neurological Institute (MNI) template*.

As expected, the task mainly activated the left primary sensorimotor areas and the right cerebellum; no artifactual cluster was observed. In both cases (1.5T and 3T), no difference was found between brain activation in the two conditions with and without glove: the result of the statistical comparison between the two conditions gave no suprathreshold clusters. In addition, thanks to the use of GAF, it was possible to quantitatively describe the finger motor task performed simultaneously with the study of brain activity; the obtained kinematics parameters are also shown in the figure.

## Discussion

In this work, we assessed the MR compatibility of an engineered glove able to record finger touches during sequences of finger opposition movements, to allow a quantitative description of finger motor performance during fMRI studies of task-related activity.

Several tests were performed by following a specific order since each of them represented a step toward the MR compatibility demonstration and they were all required to be passed to ensure no device-induced risks for safety and image artifacts, thus allowing the device use on individuals.

In fact, each test was thought to bring the device closer and closer to the scanner isocenter where the magnetic field reaches its highest level. First, with the test for magnetic attraction outside the scan room we demonstrated that the GAF system could be introduced in the MRI environment. Then, it was taken inside the scan room and tested for absence of magnetic force or torque. Third, it underwent tests of image artifacts based on the analysis of an MR Phantom representing a patient head. The tests in conjunction with the Phantom were performed in three different conditions: No GAF, GAF in FOV and GAF out of FOV.

Our findings showed that GAF did not compromise image quality of both EPI and T2 sequences when it was positioned at the level of the hand (i.e., about 70 cm far from the MRI Phantom, corresponding to the head), which represents its proper use. It should be noted that at this distance from the magnet’s isocenter, the time-varying gradient magnetic field is approximately zero.

The SNR results showed that the GAF in FOV condition was not comparable to the No GAF and GAF out of FOV results. The reason of that difference derives from the standard deviation value which increased when GAF was inside the FOV and sometimes it showed twice the values observed in the other two conditions. Statistical analysis indicated that the No GAF and the GAF out of FOV cases were not significantly apart, whereas each of the two conditions was significantly different from the GAF in FOV condition. The same conclusion was found for both T2-weighted and EPI sequences.

In addition, the Geometric accuracy test showed that the presence of GAF both in and out of FOV did not cause any significant change in the diameter value. Conversely, the Slice thickness test results were not correct if the GAF system was put into the FOV. An explanation of this finding could be that in this case GAF interferes with the radio frequency pulses of the coil. The Low-contrast object detectability test proved that if the GAF system was in the FOV, there was no possibility to distinguish any complete spoke (only very few disks were visible); when the number of disks was automatically counted, more disks with respect to their real number were detected because also some image artifacts were considered as disks by applying an intensity threshold. Thus, in the GAF in FOV condition the image quality was altered, while in the GAF out of FOV condition the result was really close to the exact number of disks.

The fMRI study showed that the use of GAF to measure motor performance simultaneously with brain activity did not alter image quality and results. Further, the finger motor parameters obtained thanks to the device were not influenced by the fMRI acquisition, but were in line with published results with similar protocols outside the MR environment (Bove et al., 2007).

Therefore the GAF system was demonstrated to be MR compatible and usable during fMRI exams. The importance of this result is that fMRI and motor tasks experiments can be taken simultaneously. It is thus possible to find out both the brain areas involved in the task execution and the spatial and temporal parameters useful to investigate the degree of precision of the sensorimotor strategy with which the motor action is performed. For instance, this makes possible to assess a patient improvement in repeating a task after undergoing rehabilitative exercises or to determine the increasing disability during the course of a neurological disease.

In conclusion, we showed that GAF was able to record finger opposition movement sequences without inducing artifacts during fMRI sessions demonstrating that GAF is compatible with the 1.5T and 3T magnetic field environment. Moreover, the simplicity of the GAF set-up allows it to become an important candidate in evaluating the sensorimotor performance of patients affected by neurological diseases during fMRI recording. Indeed, the obtained quantitative indexes describing finger opposition movements are very useful to better describe the patient’s disability status and can be correlated with structural and functional imaging parameters (Johansen-Berg, 2007; Rocca et al., 2007; Bonzano et al., 2008, 2011a,b, 2013b; Wegner et al., 2008; Ceccarelli et al., 2010; Bosnell et al., 2011; Le Bihan and Johansen-Berg, 2012; Tomassini et al., 2012; Fling et al., 2014; Pardini et al., 2014), thus allowing monitoring single patients in follow-up studies (e.g., to evaluate the effects of a pharmacological or rehabilitative treatment) or comparing groups of patients (Bonzano et al., 2013a, 2014), even in multicentric studies.

## Author Contributions

LB designed the work, acquired, analyzed and interpreted the data, wrote the manuscript. AT acquired and analyzed the data, drafted the manuscript. LR designed the work, acquired the data, drafted the manuscript. MI acquired and analyzed the data, drafted the manuscript. GLM interpreted the data and revised the manuscript. AN helped build the experimental set-up and revised the manuscript. MB designed the work and interpreted the data, wrote the manuscript. All the authors approved the final version of the manuscript.

## Conflict of Interest Statement

Antonio Novellino is part of the Research and Development Unit of ETT S.p.A. and worked on the GAF software and device development. The views expressed in this paper do not necessarily reflect the views or policy of the company, which approved the document for publication but was not involved in the study design, data collection, analysis and interpretation, or manuscript preparation. The other authors declare that the research was conducted in the absence of any commercial or financial relationships that could be construed as a potential conflict of interest.

## Footnotes

^1^http://blog.kowalczyk.info/software/volante/database.html

^2^http://www.acr.org/
